# Combined endoscopic approach with fluoroscopy-guided radial incision and cutting for a complete esophageal stricture

**DOI:** 10.1055/a-2604-7927

**Published:** 2025-06-18

**Authors:** Akito Furuta, Shunsuke Omoto, Shunsuke Ogata, Hironori Tanaka, Mamoru Takenaka, Taro Inoue, Wataru Ono

**Affiliations:** 1 145700 Department of Gastroenterology, Kobe Tokushukai Hospital, Kobe, Japan; 2 Department of Gastroenterology, Kishiwada Tokushukai Hospital, Kishiwada, Japan; 3 Department of Gastroenterology and Hepatology, Kindai University Faculty of Medicine, Osaka-Sayama, Japan

The treatment options for benign esophageal strictures include endoscopic balloon dilation (EBD), bougie dilation, and radial incision and cutting (RIC) 1 2 . RIC has been proposed for refractory cases 3 . While stricture recurrence after RIC has been reported, a multimodal approach combining RIC, EBD, and steroid therapy is suggested as a safe and effective strategy 4 5 .

This video shows a case of a complete esophageal stricture successfully treated with RIC and EBD under fluoroscopic guidance ( Video 1 ).

Complete esophageal stricture successfully managed using a combined endoscopic approach with fluoroscopy-guided radial incision and cutting, along with balloon dilation.Video 1

A 74-year-old man underwent a thoracoscopic esophagectomy for advanced esophageal cancer. On postoperative day (POD) 4, anastomotic leakage occurred and was managed with a fully covered esophageal stent. On POD 46, dysphagia developed due to stent migration, necessitating removal of the stent. Oral intake became impossible and repeated EBD was followed by a poor response. RIC was initiated on POD 86.

On the day of RIC, the stricture had progressed to the extent that the lumen was unidentifiable, and it was assessed as complete stricture ( Fig. 1 ). An initial incision with a flush knife BT-S 2.0 (Fujifilm Medical, Tokyo, Japan) was attempted, but due to the risk of perforation, fluoroscopic guidance was employed. A guidewire and catheter were advanced beyond the stricture to facilitate a safer incision ( Fig. 2 ). Incision and bougienage were alternated, followed by an arc-like IT knife (Olympus, Tokyo, Japan) incision to remove the scar tissue ( Fig. 3 ). The stricture was then dilated with a balloon, allowing the passage of an endoscope ( Fig. 4 ).

**Fig. 1:**
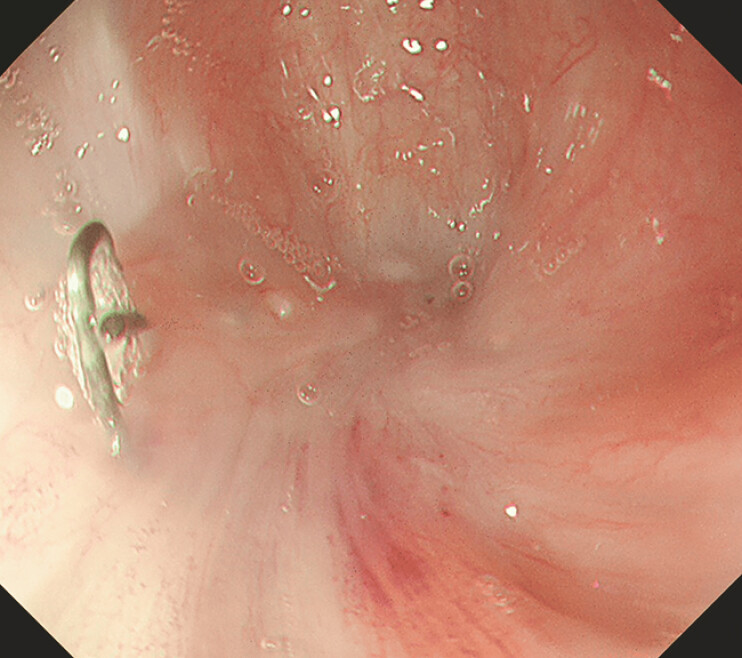
On the day when radial incision and cutting (RIC) was carried out, the stricture had progressed to the extent that the lumen was unidentifiable, and it was assessed as complete stricture.

**Fig. 2:**
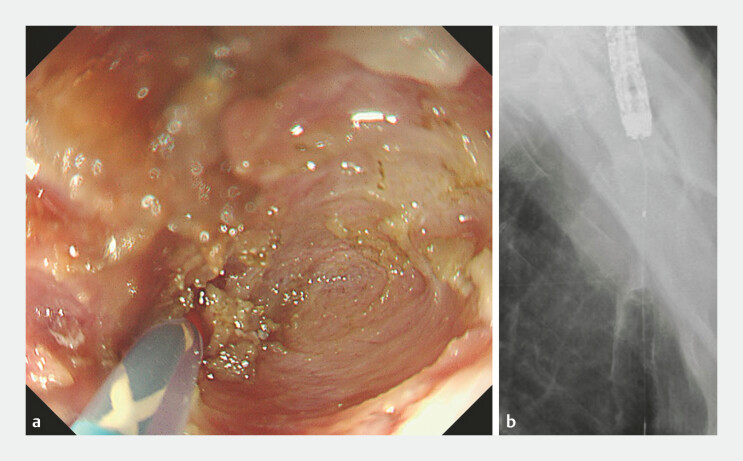
**a, b** Under fluoroscopic guidance, a guidewire and catheter were advanced beyond the stricture to facilitate a safer incision.

**Fig. 3:**
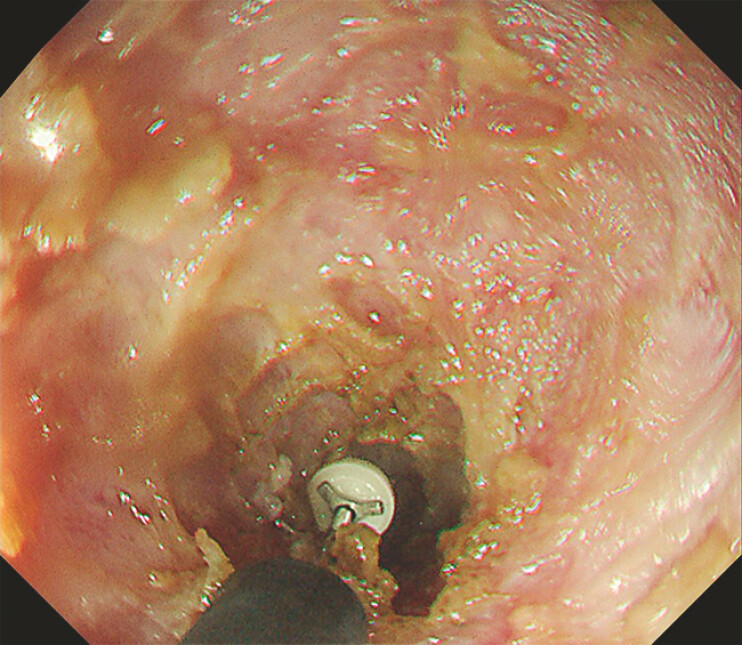
After advancing the incision to a certain extent, a switch was made to an IT knife and the scar tissue was removed in an arc-like manner.

**Fig. 4:**
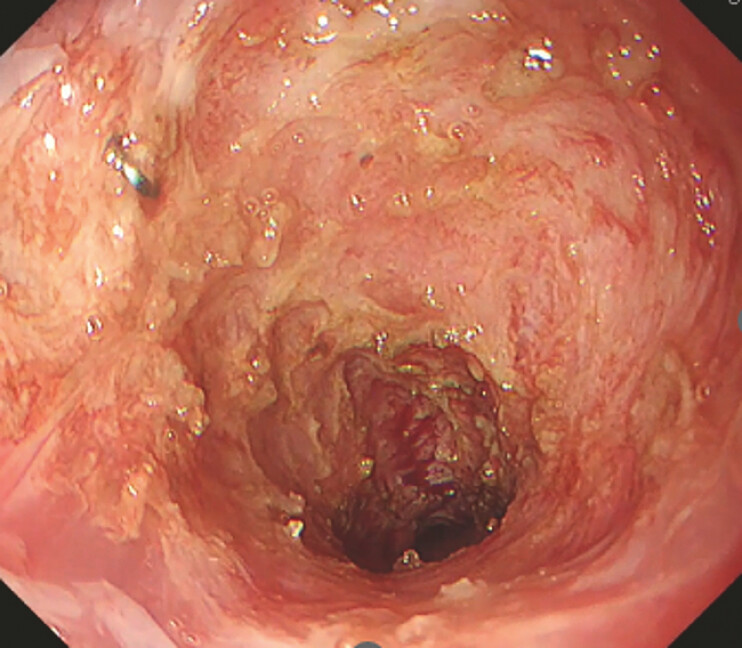
The stricture was successfully dilated, allowing the passage of an endoscope.

The patient underwent repeated EBD and an additional RIC followed by an injection of 40 mg triamcinolone acetonide. Oral prednisolone therapy was subsequently initiated at a dose of 30 mg per day. Six months after the initial RIC, he was able to consume almost all solid foods without difficulty or stricture recurrence ( Fig. 5 ).

**Fig. 5:**
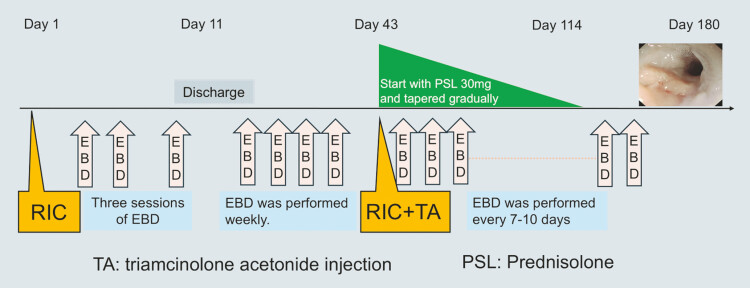
The patient underwent repeated endoscopic balloon dilation and an additional RIC, followed by steroid therapy. Six months after the initial RIC, he showed significant improvement, being able to consume almost all solid foods without difficulty and without stricture recurrence.

This case demonstrates that a comprehensive multimodal approach combining fluoroscopy-guided RIC, EBD, and steroid therapy can provide a safe and effective treatment strategy for complete esophageal strictures.

Endoscopy_UCTN_Code_TTT_1AO_2AH

## Notes

Endoscopy E-Videos https://eref.thieme.de/e-videos
*E-Videos* is an open access online section of the journal *Endoscopy* , reporting on interesting cases and new techniques in gastroenterological endoscopy. All papers include a high-quality video and are published with a Creative Commons CC-BY license. Endoscopy E-Videos qualify for HINARI discounts and waivers and eligibility is automatically checked during the submission process. We grant 100% waivers to articles whose corresponding authors are based in Group A countries and 50% waivers to those who are based in Group B countries as classified by Research4Life (see: https://www.research4life.org/access/eligibility/ ). This section has its own submission website at https://mc.manuscriptcentral.com/e-videos .
